# Of Tidal Waves and Human Frailty

**DOI:** 10.3201/eid1110.AC1110

**Published:** 2005-10

**Authors:** Polyxeni Potter

**Affiliations:** * Centers for Disease Control and Prevention, Atlanta, Georgia, USA

**Keywords:** Art and science, emerging infectious diseases, Katsushika Hokusai, Mt. Fuji, The Great Wave

**Figure:**
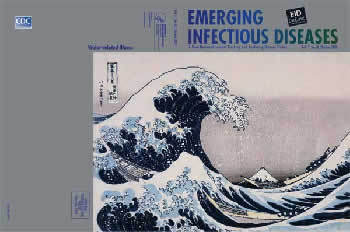
Katsushika Hokusai (1760–1849). Thirty-six Views of Mt. Fuji: The Great Wave off Kanagawa (1830–2) (detail). Color and ink on paper (25.7 cm × 37.9 cm). Honolulu Academy of Arts, Hawaii, USA, Gift of James A. Michener, 1991 (13,675)

From the age of six I had a penchant for copying the form of things, and from about fifty, my pictures were frequently published; but until the age of seventy, nothing that I drew was worthy of notice. At seventy-three years, I was somewhat able to fathom the growth of plants and trees; and the structure of birds, animals, insects and fish. Thus when I reached eighty years, I hope to have made increasing progress, and at ninety to see further into the underlying principles of things, so that at one hundred years I will have achieved a divine state in my art, and at one hundred and ten, every dot and every stroke will be as though alive.

From Hokusai's autobiography, written in 1835, at age 75

"The old man mad about painting" was how Katsushika Hokusai signed some of his work in his later years (*1*). Passion for art defined his life. And on his deathbed, at age 89, he bemoaned, "If only Heaven will give me just another ten years… just another five more years, then I could become a real painter" (*1*).

Hokusai was born in Edo, present-day Tokyo. He showed early interest in art and was apprenticed to Katsukawa Shunshō, master painter and printmaker, to paint *ukiyo*-*e*, "images of the floating world," a style focused on everyday activities and their fleeting nature. He painted the transient lives of actors in Edo's theater district, then moved on to study other art styles and become famous for his illustrations of poetry and popular novels. He drew from diverse artistic traditions, among them Chinese and Western art, which was then beginning to appear in Japan. Versatile and prolific, he left thousands of works, signed in more than 30 artistic names. He created a series of sketchbooks as instruction to those who wanted to draw in his style. The series was called Hokusai manga, a term he coined (*2*).

In a traditional society of Confucian values and rigid regimentation, Hokusai was bohemian. Eccentric, rebellious, and temperamental, he cared nothing about convention and was reputed to move each time the notorious clutter and disorder of his home became unbearable. Legend has it that when invited once to paint maple leaves floating on the Tatsuta River, he drew a few blue lines and then repeatedly imprinted atop the scroll chicken's feet he had dipped into red color. When his contemporaries drew the shoguns and samurai, he portrayed the common people, and when he painted landscapes, it was strictly from his own point of view (*3*).

Even though Hokusai's work did not receive full appreciation in Japan, it gained high status and respect abroad. The Great Wave (on this month's cover) became a global icon, as recognizable and revered as Leonardo da Vinci's Mona Lisa or Vincent van Gogh's Sunflowers. Hokusai prints were collected by Claude Monet, Edgar Degas, Mary Cassatt, and many others, who were influenced by them.

Hokusai reached the peak of his creativity in his seventies, when he began work on his thirty-six views of Mount Fuji (3,776 m), Japan's summit and spiritual epicenter. These images, like much of his mature work, reflect familiarity with such European trends as innovative pigments and the telescope. Fascinated by Western design principles, he integrated them with Japanese technique, not only in landscape paintings but also with flowers and birds, which he showed in horizontal close-ups and cut-outs as if seen by a telescope. His imaginative efforts captured the essence rather than the likeness of what he painted and created an altogether novel effect, which appeared Japanese to outsiders and Western to the Japanese.

The Great Wave is Hokusai's most celebrated work. Although renowned nature scenes featured often in Japanese art, the landscape as *ukiyo*-*e* theme did not gain prominence until after views of Mount Fuji prints became popular. The Great Wave inspired other artistic works, as diverse as Rainer Maria Rilke's poem Der Berg (The Mountain) and Claude Debussy's symphonic masterpiece La Mer (The Sea), whose full score featured The Great Wave on its first edition at the request of the composer (*4*).

This refined woodblock print epitomizes the artist's skills. Although meticulously structured, it appears effortless, its flair equaled only by the purity of its composition. Undulating lines are fine, at times almost invisible, the colors deliberate and intense. The viewer is guided through the perilous ebb, past the boats to the landmark mountain. The wave is menacing and ghostly, hardened by thick skeletal lines, softened by bubbles of mist, sparkling and voluminous. An eerie feeling is punctuated by the pale sky and frosty white of breaking waves and mountain peak.

The scene could not be more *ukiyo*-*e*: three light boats carrying fish to market on a work day. But on this day, the sea is in charge, a monstrous wave commanding the foreground, cresting high above the horizon, dwarfing majestic Mt. Fuji now a bump in the fluid scene. Like leaves tossed to sea, the boats tumble, their tiny occupants crouched in fear, clinging to the sides, unable to face the wave and its claws of foam curling toward them.

In The Great Wave, Hokusai captured the uneasy sentiments of a nation surrounded and defined by water, as well as the deeper, primal, human terror of the sea. Enchanting but treacherous, water lures and repels. Seeking livelihood, fortune, adventure, or just solace in its calm, humans ride the waves, risking capricious tempests, settling in precarious coastal regions frequently battered and overpowered by the sea. When the earth moves or climate and other elements stir the waters, environmental markers shift, boats and settlements crumble, and humans perish. In the aftermath comes infectious disease, originating in the disruption and lingering for lack of hygienic conditions and adequate medical care.

Hokusai's fishermen typify human plight against overwhelming force. Their posture embodies the horror of imminent physical harm and death. Fear and anxiety about the long-term consequences of environmental catastrophe are left to survivors and public health workers, who face, along with the loss of infrastructure, compromised sanitation, contamination of water supplies, secondary wound infections, unsafe food, increased poverty, and compounded disease.

The formidable challenge of water-related illness and death persists, from the Indian Ocean to the Gulf of Mexico—despite global prevention and control efforts. Like the fishermen caught in Hokusai's wave, unable to confront the culprit, we cling to a lifeline: managing the physical trauma and addressing resultant infections and complications.
